# Patient-centric characterization of multimorbidity trajectories in patients with severe mental illnesses: A temporal bipartite network modeling approach

**DOI:** 10.1016/j.jbi.2022.104010

**Published:** 2022-03

**Authors:** Tao Wang, Rebecca Bendayan, Yamiko Msosa, Megan Pritchard, Angus Roberts, Robert Stewart, Richard Dobson

**Affiliations:** a Department of Biostatistics and Health Informatics, King’s College London, Denmark Hill, London SE5 8AF, United Kingdom; b National Institute for Health Research, Maudsley Biomedical Research Centre, South London and Maudsley National Health Service (NHS) Foundation Trust, Denmark Hill, London SE5 8AZ, United Kingdom; c Department of Psychological Medicine, King’s College London, Denmark Hill, London SE5 8AF, United Kingdom; d Institute of Health Informatics, University College London, Euston Road, London NW1 2DA, United Kingdom; e Health Data Research UK London, University College London, Euston Road, London NW1 2DA, United Kingdom

**Keywords:** Multimorbidity, Severe mental illnesses, Temporal bipartite network, Disease trajectories, EHR data linkage, Network evolution

## Abstract

Multimorbidity is a major factor contributing to increased mortality among people with severe mental illnesses (SMI). Previous studies either focus on estimating prevalence of a disease in a population without considering relationships between diseases or ignore heterogeneity of individual patients in examining disease progression by looking merely at aggregates across a whole cohort. Here, we present a temporal bipartite network model to jointly represent detailed information on both individual patients and diseases, which allows us to systematically characterize disease trajectories from both patient and disease centric perspectives. We apply this approach to a large set of longitudinal diagnostic records for patients with SMI collected through a data linkage between electronic health records from a large UK mental health hospital and English national hospital administrative database. We find that the resulting diagnosis networks show disassortative mixing by degree, suggesting that patients affected by a small number of diseases tend to suffer from prevalent diseases. Factors that determine the network structures include an individual’s age, gender and ethnicity. Our analysis on network evolution further shows that patients and diseases become more interconnected over the illness duration of SMI, which is largely driven by the process that patients with similar attributes tend to suffer from the same conditions. Our analytic approach provides a guide for future patient-centric research on multimorbidity trajectories and contributes to achieving precision medicine.

## Highlights

• Temporal bipartite networks offer a natural representation for diagnosis data.
• Health data linkage provides comprehensive disease profiles for patients.
• Patients with similar attributes tend to be diagnosed with the same diseases.
• Patients with the same disease have heterogeneous disease processes.
• Shorter distances of patient and disease nodes over time reveal disease processes.

## Graphical abstract

**Figure:**
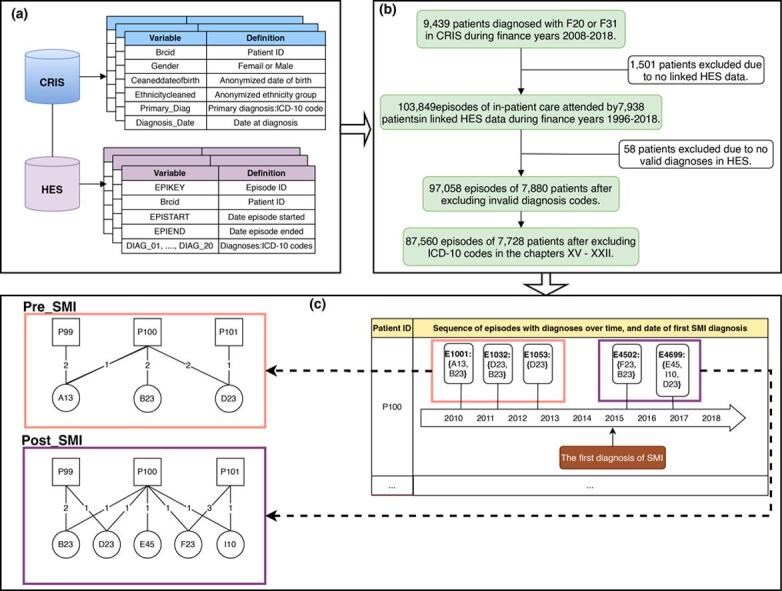

## 1 Introduction

Patients with severe mental illnesses (SMI), such as schizophrenia and bipolar affective disorder, have increased mortality rates compared to the general population, with a 10–20 year reduction in life expectancy [1], [2], [3]. Although deaths due to suicide and violence contribute to these excess mortality rates, a majority (approximately two-thirds) of premature deaths in patients with SMI have been attributed to physical comorbidities, such as cardiovascular disease, smoking-related lung disease or type 2 diabetes [4], [3]. In fact, multimorbidity, defined as co-occurrence of two or more health conditions, is common among patients with SMI [3], [5]. This makes these patients especially challenging and costly to manage, because (1) existing clinical guidelines largely focus on managing a single disease and rarely deal with multimorbidity [6], [7] and (2) these patients are more likely to be vulnerable to the adverse consequences of transitions in care provision for different conditions [8]. Thus, increasing attention has focused on early detection and management of multimorbidity, particularly on physical health conditions, to improve health outcomes and reduce premature mortality for patients with SMI [9]. To effectively prevent and detect potential future diseases, a key prerequisite is a deep understanding of when and how different diseases occur and interact during an individual’s life course.

Addressing this question requires longitudinal healthcare data for patients with SMI and appropriate statistical methods for modeling temporal interactions of diseases, considering illness duration and other relevant risk factors such as age, sex and ethnicity [10], [11], [12], [13]. Prior research on multimorbidity has focused on cross-sectional studies which examine prevalence of multimorbidity in populations sampled within various settings, e.g., different geo-locations, sources of data or periods of time [4], [14], [15], [16], [3], [17], [18], [19], [20]. There are only a few longitudinal studies which have aimed to understand how conditions appear across the lifespan of an individual with SMI [21], and this is partly because of lack of longitudinal healthcare data for this population. Among these studies, some used routine data collected from a local healthcare provider [4], [3] or others were based on national administrative claims data [18], [19]. Routine data are often reliable to identify likely cases [22] but may miss care information from other healthcare providers, which can lead to an incomplete observation of a patient’s medical history. In contrast, administrative datasets may contain a more comprehensive medical history for a patient but tend to under-record psychiatric diagnoses [23], [22], [24], which can lead to a biased sample. So far, there has been no large-scale studies which link both types of data sources to gain a more comprehensive understanding of disease profiles in patients with SMI. Although recent studies have estimated the impact of a disease on mortality in patients with SMI based on retrospective cohort data using regression analyses [25], [5], these analytical methods are limited in their ability to capture correlations among diseases over time [26]. It therefore remains unclear how different diseases interact with one another over the lifetime of a patient with SMI.

Recent evidence has shown that network models provide a powerful means to characterize interactions among diseases [27], [28], [29] and explore temporal progression trajectories for complex conditions such as diabetes and cardiovascular diseases [10], [30], [31], [11], [32]. These methods often represent patient-diagnosis data as a one-mode network, in which each node represents a disease and an edge links two nodes if two diseases have a strongly statistical correlation, such as co-occurrences [33], [29], [32] and sequential associations [10], [11], which enables us to examine relationships of diseases/symptoms, e.g., disease progression paths, by measuring structural properties of these networks. Despite such advances, existing models are dominated by disease-centric approaches, i.e., focusing on exploring the relationships of diseases at a population level and ignoring different attributes of individual patients. However, previous studies have shown that each individual patient may develop a distinct pathway in disease development [34] as a wide range of individuals’ attributes e.g. age, gender, ethnicity and the order of incidence, are important factors contributing to disease progression [35], [30]. It is vital to take individual patients and their attributes into account when investigating the relationships between diseases. Moreover, as we elaborate below, ignoring heterogeneity in individual patients – by looking only at aggregates across a whole cohort – can lead to a misleading estimate of a disease relationship.

In this study, we present an alternative approach, namely temporal bipartite networks, to characterize time-dependent multimorbidity profiles for patients with SMI based on a large set of linked longitudinal healthcare data. We leverage electronic health records (EHRs) from the South London and Maudsley (SLaM) National Health Service (NHS) Foundation Trust, one of the largest secondary mental health care providers in the UK, to identify a large cohort of patients with SMI and link to Hospital Episode Statistics (HES) data [36], an English national hospital administrative database, to collect records of all admitted care of these patients across NHS hospitals in England. Based on this unique dataset, the main objective of this study is to achieve a systematic investigation of the changes in diagnoses among patients with SMI over time from both disease-centric and patient-centric perspectives using temporal bipartite networks.

## 2 Material and methods

In this retrospective cohort study, we analyzed a linked dataset between secondary mental-health care data from the SLaM NHS Foundation Trust and longitudinal admitted-patient care data from the HES dataset. SLaM is one of the largest mental health trusts in the UK, providing a wide range of secondary mental health services for 1.3 million residents in South London and specialist services for people across the UK. HES data contain details of all admitted patient care at NHS hospitals in England [36], and have been routinely collected by a national service since 1996 [37]. All data were collected through the Clinical Record Interactive Search (CRIS) system, a psychiatric case register which allows authorized researchers to explore fully de-identified EHRs in SLaM [38], [39]. CRIS has been linked with the HES data for all patients who have used SLaM services, regardless of where they were living at the time of their hospital use [40]. Diagnoses in both CRIS and HES data were coded using 3/4-character codes from the International Classification of Diseases, 10th revision (ICD-10). This study has been approved by the CRIS Oversight Committee, under an ethical approval for secondary analysis of anonymized data from the Oxford Research Ethics Committee (reference 06/H0606/71+5) [41].

### 2.1 Study design and data collection

**Fig. 1:**
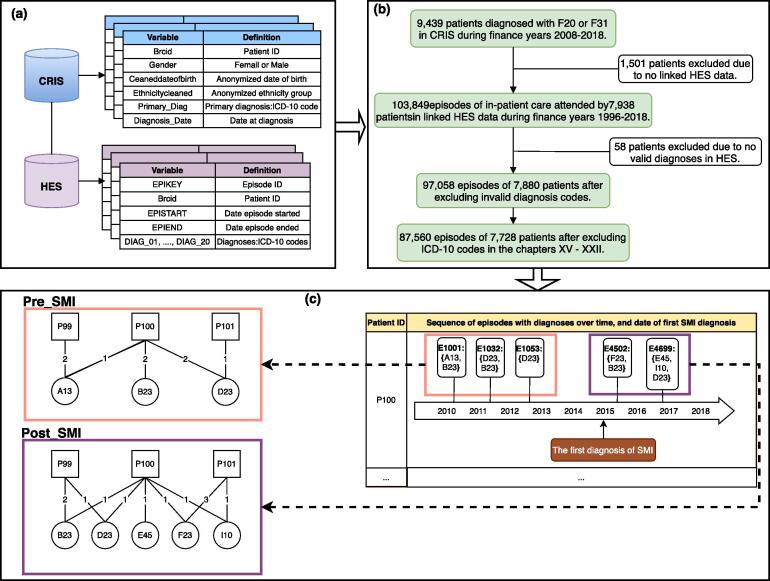
Study design and data flow. (a) Key variables from CRIS and HES databases used in data collection. (b) Flow chart of cohort selection for SMI patients and diagnosis code selection. (c) Network representations of patients’ diagnoses in pre-SMI and post-SMI periods. An un-directed edge links a patient node (denoted by a square) and a disease node (denoted by a circle) if the patient was diagnosed with the disease, weighted by the frequency of such diagnoses.

A data-driven approach was used in this study to characterize disease trajectories for patients with SMI. Fig. 1 shows our study design and data flow. We first identified a cohort of patients who had a primary diagnosis of SMI from April 1, 2008 to March 31, 2018 in CRIS, particularly focusing on two of the most common SMI: schizophrenia (with the ICD-10 code of “F20”) and bipolar disorder (“F31”) [42]. For each patient, we collected their (1) demographic information such as date of birth, gender and ethnicity, and (2) diagnostic information including the first diagnoses of SMI and dates of diagnoses. Second, we gathered all admitted-patient episodes from April 1, 1996 to March 31, 2018 for the cohort in the linked HES data. Each HES record comprises up to 20 primary and secondary diagnoses, and dates of admission and discharge. We only included HES records with at least one valid diagnosis, and excluded non-disease diagnoses with an ICD-10 code in chapters XV-XXII [13]. For patients who were diagnosed with SMI in other NHS trusts before the diagnosis was recorded in SLaM, we identify their first SMI diagnosis and the diagnosis date by selecting the earliest SMI diagnosis across their CRIS and HES records.

### 2.2 Temporal bipartite networks

As shown in Fig. 1c, we represent patient-diagnosis data using temporal bipartite networks and characterize patients’ disease trajectories by measuring structural differences of these temporal networks. Given a collection of admission episodes *C* over a time period [0,T], a temporal bipartite network $G_{i}$ at timestamp $t_{i}(i=1,2,\ldots ,n,0⩽t_{i}⩽T)$ can be defined as:

Definition 1 A temporal bipartite network $G_{i}=(U,V,E_{i})$ is a graph that consists of two disjoint sets of nodes, *U* and *V*, representing patients and diseases respectively, and a set of temporal edges $E_{i}=\{(u,v,w)|u\in U,v\in V,\exists (u,v,t_{i})\in C\}$, such that an un-directed edge $(u,v)\in E_{i}$ in $G_{i}$ links a patient node *u* and a disease node *v* if the patient was diagnosed with the disease at time $t_{i}$, weighted by the frequency of such diagnoses *w*.

In each network $G_{i}$, we represent patients’ characteristics by (1) encoding gender, ethnicity, the first SMI diagnosis and age at the first SMI diagnosis as attributes of patient nodes, and (2) encoding age when a patient was for the first time diagnosed with a disease as an attribute of an edge. Following previous studies [30], [43], [13], all ICD-10 codes were rounded to 3 characters as the first 3 characters capture the main category of a diagnosis. Unlike one-mode network models that merely capture relationships among a single type of nodes (e.g., diseases) [33], [29], [30], [28], [31], [11], [32], the proposed bipartite network models jointly represent information on both patients and diseases, as well as their relationships, which provides a natural representation and preserves more information in patient-diagnosis data.

### 2.3 Temporal network analyses

We start our analyses of temporal networks from a lower temporal resolution and examine overall differences in patients’ diagnoses during hospital admissions before and after their first diagnoses of SMI. We split a patient’s admission episodes into pre- and post-SMI subsets based on the date when the patient was first diagnosed with SMI and the admission dates of HES episodes. Patients’ diagnoses in each subset are then represented using a bipartite network. For clarity, the resulting pre-SMI and post-SMI networks are aggregated bipartite networks, defined as:

Definition 2 An aggregated bipartite network $G_{i,j}=(U,V,E_{i,j})$ is a graph that includes all episodes and related temporal edges between nodes in period [$t_{i},t_{j}$], where $E_{i,j}=\{(u,v,w)|u\in U,v\in V,\exists (u,v,t_{i},t_{j})\in C\}$.

We then extend the temporal resolution from a binary scale (i.e., pre- and post-SMI periods) to a multilevel scale (e.g., years to the first SMI diagnoses) to explore the process of network evolution in detail. For each episode, we calculate the number of years $t_{i}$ from the date that an episode was recorded to the date that a patient was first diagnosed with SMI, where pre-SMI episodes have a negative value of $t_{i}$. Then, a sequence of temporal bipartite networks are created based on a snapshot of data at time period $t_{i}$. Due to a small number of episodes in a single-year period, we use a sliding window to address the issues of data sparsity. Specifically, given a sequence of episodes over *n* periods $\left\{t_{1},\ldots ,t_{n}\right\}$, the snapshots of bipartite networks are defined as:

Definition 3 Snapshots of bipartite networks are a sequence of graphs denoted by $\left(G_{t_{1},t_{1}+\Delta t},G_{t_{2},t_{2}+\Delta t},\ldots ,G_{t_{i},t_{i}+\Delta t}\right)$, where $G_{t_{i},t_{i}+\Delta t}$ is an aggregated bipartite network built based on the sequence of episodes in time window $\left[t_{i},t_{i}+\Delta t\right]$ (where Δt is the size of each window, 1⩽i⩽n and $t_{i}+\Delta t⩽t_{n}$).

Note that, to achieve more meaningful comparisons between temporal networks^1^, we excluded edges on a patient’s first SMI diagnosis in network construction and encoded a patient’s first SMI diagnosis as an attribute of the patient node. For example, if a patient was first diagnosed with “F31” and then diagnosed with “F20” at a later stage, “F31” is used as an attribute of the patient node and all “F31” diagnoses of this patient are excluded in the post-SMI networks, while “F20” diagnoses are included in the post-SMI networks. Thus, a patient who only had a SMI diagnosis and did not have diagnoses on other diseases were not included in the network models.

Also, for ease of presentation, we denote conditions that appear in pre-SMI periods as *pre-existing conditions*, those that do not appear in pre-SMI periods and only appear in the post-SMI periods as *new conditions*, and those that appear in both pre- and post-SMI periods as *re-occurring conditions*. Formally, given a patient node $u,N_{1}(u)$ denotes the set of *u*’s direct neighbors in the pre-SMI network (i.e., pre-existing conditions) and $N_{2}(u)$ denotes the set of *u*’s direct neighbors in the post-SMI network. Then, new conditions are identified as $N_{2}(u)⧹N_{1}(u)$, re-occurring conditions are identified as $N_{1}(u)\cap N_{2}(u)$. The ratio of new conditions in all conditions is computed as:

(1) $$\frac{\left|N_{2}(u)⧹N_{1}(u)\right|}{\left|N_{2}(u)\cup N_{1}(u)\right|}\text{.}$$

### 2.4 Measuring clustering tendency

We measure nodes’ distances in temporal bipartite networks to examine clustering tendency of nodes over time. The distance can be quantified by the mean length of the shortest paths between node pairs in a network: $\ell =\frac{1}{N(N-1)}\sum_{i\neq j}d_{\mathrm{ij}}$, where *N* is the number of nodes in a network and $d_{\mathrm{ij}}$ is the shortest path length from node *i* to node *j*. The definition of ℓ however is problematic if a network is not connected [44]. In these cases, some node pairs have no connecting path and hence have infinite values of *d*, which leads to an infinite value of ℓ. To avoid this issue, we measure the mean of the reciprocal of *d*, known as the average efficiency of a network [45], which is computed as: $E=\frac{1}{N(N-1)}\sum_{i\neq j}\frac{1}{d_{\mathrm{ij}}}$, where infinite values of $d_{\mathrm{ij}}$ have no contribution to the sum. The value of *E* is inversely proportional to the shortest distance ℓ, i.e., a higher value of efficiency indicates shorter distances between node pairs in a network.

### 2.5 Detecting temporal graphlets

The above methods on network evolution analysis have mainly relied on aggregating temporal information to a sequence of snapshots. These approaches however cannot fully capture temporal information in the data, e.g., the ordering of edges if the edges occur in the same snapshot. To better understand the process of network changes, we examine graphlets in temporal bipartite networks. Graphlet analysis is originally used to characterize fundamental topological patterns and uncover structural principles in static networks [48], [47], [49]. In static networks, graphlets are defined as small, connected, induced sub-graphs of a complex network; each graphlet describes a particular topology of interactions among nodes, serving as a building block in a network [50], [46], [51]. Extending to temporal networks, graphlets are often defined as small, connected, induced sub-graphs formed by a sequence of temporal edges that occur in a time window Δt [52]. Temporal graphlets capture not only the topological structure of a sub-graph, but also the temporal ordering of edges in the sub-graph, which provides an effective means for uncovering fine-grained patterns of network evolution. Formally, temporal graphlets in bipartite networks are defined as:

Definition 4 An *n*-node, *m*-edge, Δt-temporal graphlet in a bipartite network $G=(U,V,E_{\Delta t})$ is a sequence of *m* temporal edges, $D=(u_{1},v_{1},t_{1}),\ldots ,(u_{i},v_{j},t_{m})$ within a time interval Δt, where $u_{i}\in U,v_{j}\in V,t_{1}<t_{2}<\ldots <t_{m}$ and $t_{m}-t_{1}⩽\Delta t$, such that the included static sub-graph from the edges is connected and has *n* nodes.

**Fig. 2:**
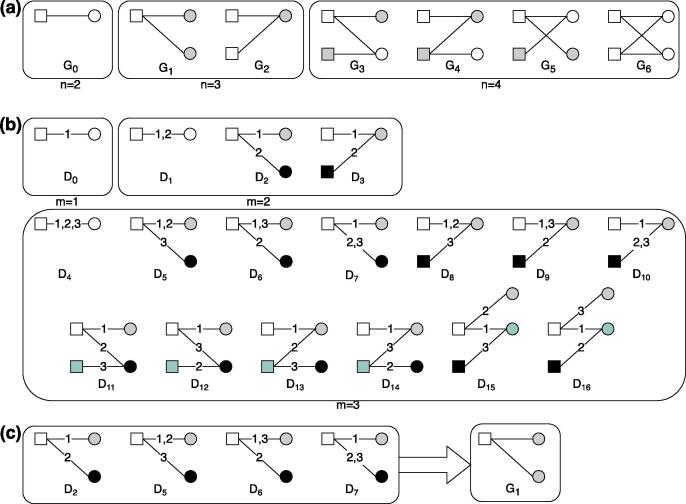
Static and temporal graphlets in bipartite networks. (a) All 7 static graphlets with n=2,3,4 nodes, where different node colors mark different automorphism orbits [46], [47], i.e., nodes’ symmetry groups. For example, there is a single orbit in graphlet $G_{6}$, as all 4 nodes are topologically identical. In contrast, there are two orbits in $G_{1}$, as the two circle nodes are topologically identical to each other but not to the square node. We here distinguish nodes’ orbits merely based on their topological positions in a sub-graph, regardless of their types. (b) All temporal graphlets with m=1,2,3 temporal edges, where an edge label marks the ordering of the edge and multiple appearances of the same edge are separated with commas. In practice, only a sequence of edges occurring within a time window Δt is considered. Different node colors denote different orbits. (c) All 4 temporal graphlets $D_{i}$ have the same structure as static graphlet $G_{1}$.

For illustration, Fig. 2 shows examples of graphlets with up to 4 nodes in static and temporal bipartite networks.

As graphlet detection is computationally expensive and its computational complexity grows substantially with increases in network and graphlet sizes (in terms of node and edge counts) [46], [47], [52], here we focus on examining temporal graphlets with 2 temporal edges. Also, since most existing algorithms for detecting temporal graphlets are developed in the context of one-mode networks [52], [49], we adopt the algorithmic framework for one-mode networks from [52], given its high efficiency and widespread use [49], and modify its counting components to fit the definition of temporal graphlets in bipartite networks. We use this adjusted algorithm to compute the counts of 2-edge, Δ-temporal graphlets in the patient-diagnosis networks. All diagnoses that a patient had in our data, i.e., including patients’ first SMI diagnoses, were included in the graphlet analysis to avoid information loss in diagnosis sequences.

### 2.6 Significance tests on network properties

To assess whether a network property, such as degree correlations, is significantly different from that expected by chance, we compare the empirical value of a network property in an original network against a null model [53]. The null model generates random networks by randomly shuffling of connections of the two parities in the original bipartite networks and preserving their degree sequences [54], [55]. For example, given two edges A-X and B-Y, we get A-Y and B-Y by swapping the two edges. This comparison distinguishes features accounted for by the degree sequence from those that might reflect other factors. The statistical significance of a network property ϕ is assessed through a *z*-score: $z=\frac{\phi_{o}-\langle \phi_{r}\rangle }{\sigma (\phi_{r})}$, where $\phi_{o}$ is the empirical value of a network property measured from an original network, $\langle \phi_{r}\rangle$ is the average value of the network property in random networks, and $\sigma (\phi_{r})$ is the standard deviation in random networks. *P*-values of *z*-scores are calculated based on two-tailed tests.

## 3 Results

We identified a cohort of 7,728 patients with a primary diagnosis of schizophrenia or bipolar disorder, where 4,636 (60%) patients were first diagnosed with schizophrenia (“F20”) and the rest 3,092 patients were first diagnosed with bipolar (“F31”). The mean age when patients were first diagnosed with “F20” is 39.5 with a standard deviation (SD) of 16.7, and that diagnosed with “F31” is 39.4 (SD = 16). No significant difference was found between two groups in their age distributions (P=0.88). See supplemental information (*SI*) for descriptive statistics of patient demographics and admission episodes. Table 1 shows descriptive statistics of the pre- and post-SMI networks, as well as the network built on episodes across all periods. A general feature of these networks is sparse connections among nodes, i.e., a low likelihood that two nodes are connected by a single link, as indicated by the small values of density. However, most nodes (more than 99.7%) in a network are connected in one giant component, meaning that almost any two nodes are reachable from one another through links. Next, we examine the pre- and post-SMI networks in detail to explore differences in patients’ diagnoses during hospital admissions before and after SMI diagnoses.

**Table 1:** Characteristics of networks, including numbers of nodes (#nodes), numbers of patient nodes (#patients), numbers of disease nodes (#disease), numbers of edges (#edges), average degrees of all nodes (〈k〉), average degrees of patient nodes ($\langle k_{P}\rangle$), average degrees of disease nodes ($\langle k_{D}\rangle$), density which is the ratio of the number of edges to the number of possible edges in a network given by #patients×#diseases, numbers of connected components (#Comps) and ratios of nodes in the giant/largest connected component (%GCR).

Time	#Nodes	#Patients	#Diseases	#Edges	〈k〉	$\langle k_{P}\rangle$	$\langle k_{D}\rangle$	Density	#Comps	%GCR	
Pre-SMI	5,680	4,928	752	18,750	6.6	3.8	24.93	$5.06\times 10^{-3}$	9	99.72	
Post-SMI	6,332	5,535	797	40,931	12.93	7.39	51.36	$9.28\times 10^{-3}$	5	99.86	
All	7,964	7,116	848	55,321	13.89	7.77	65.24	$9.17\times 10^{-3}$	2	99.97	

### 3.1 Disease-centric analysis

We first analyze our results from a disease-centric preservative, particularly focusing on examining which diseases are often diagnosed before and after the first diagnosis of SMI, whether pre- and post-SMI diagnoses differ from each other, and which factors are associated with these differences.

#### 3.1.1 Time-dependent multimorbidity profiles

As shown in Table 1, the average degree of patient nodes $\langle k_{P}\rangle$ is 3.8 and 7.39 in the pre- and post-SMI networks, i.e., a patient on average suffered from 3.8 and 7.39 conditions in pre- and post-SMI periods respectively. By further examining the degree distributions of patient nodes (see Fig. S3b in *SI*), we find that 45.4% (N=3,235) and 63.7% (N=4,534) patients have more than one disease (i.e., $k_{p}⩾2$) in the pre- and post-SMI networks respectively. These results imply that most patients with SMI experience multimorbidity, i.e., co-existence of two or more medical conditions [56], [29]. However, a patient’s pre-SMI and post-SMI episodes can span across years (see Fig. S2e in *SI*) and people can have a variety of conditions at different stages of life. It may not be surprising that a patient node links to more than one disease node in these networks. To avoid over-estimates of multimorbidity due to long time periods covered in the networks, we conduct a sensitivity analysis by only including patients’ episodes within a relatively short time window before and after their SMI diagnoses respectively. We find that 36.6% (N=2,603) and 52.9% (N=3,764) of patients have more than one health condition within a 5-year window before and after the date of the first SMI diagnosis respectively.

Moreover, some patients may be diagnosed with other mental health conditions due to inaccurate diagnosis during the process of SMI diagnosis rather than due to multimorbidity. For example, bipolar disorders are often mis-diagnosed and initially treated as some of their severe symptoms such as psychosis [57], which can lead to over-report of multimorbidity for patients with bipolar. To eliminate such effect, apart from excluding episodes outside the 5-year windows, we further exclude mental health diagnoses (identified by the ICD-10 codes starting with “F”) in building pre-SMI and post-SMI networks for another sensitivity check. We find that 23% (N=1,640) and 38.6% (N=2,744) of patients have more than one physical health condition within a 5-year window before and after the date of the first SMI diagnosis respectively (see *SI* for the detailed lists of the most common conditions and their incidence timing in our cohort). These results confirm that multimorbidity is common in patients with SMI.

**Fig. 3:**
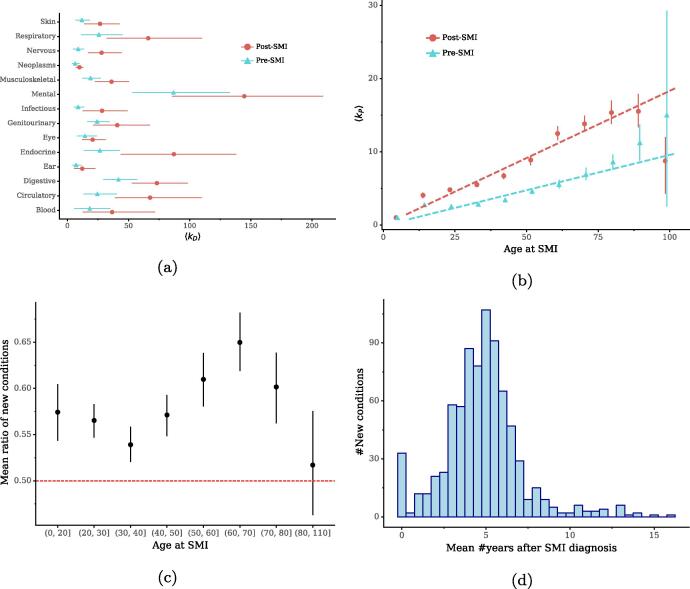
(a) Average degrees of disease nodes by the ICD-10 categories/chapters. (b) Average degrees of patient nodes by age at the first SMI diagnosis, where dashed lines plot correlations between age at SIM diagnosis and $\langle k_{P}\rangle$ by a linear regression model. (c) Ratios of new diseases over age at SMI diagnosis, where the red horizontal marks the cutoff of 0.5. (d) Distribution of new conditions by the average number of years from the dates of patients’ first SMI diagnoses to the dates of their first diagnosis of a condition. Error bars in all plots indicate 95% CIs.

To understand which are the most common comorbidities among patients with SMI, we group conditions by the ICD-10 chapters and examine the degree distributions of diseases in each chapter (Fig. 3a). We find that endocrine, respiratory, digestive and circulatory diseases are the most common comorbidities among these patients. Moreover, conditions in each chapter on average have a higher degree in the post-SMI network than in the pre-SMI network, suggesting increased prevalence of conditions in post-SMI periods, particularly for nervous and endocrine diseases. For detailed lists of prevalent conditions in pre- and post-SMI periods, as well as analyses of multimorbidity profiles in patients with different types of SMI, See *SI*.

#### 3.1.2 Multimorbidity progression

Another finding in Table 1 is that the average degree of patient nodes $\langle k_{P}\rangle$ in the post-SMI network is higher than that in the pre-SMI network. This indicates that patients on average suffer from a larger number of conditions after their SMI diagnoses. To explore whether this increase of number of condition after SMI diagnoses is associated with normative ageing, we stratify patients by their age at the first diagnosis of SMI and examine the average degrees in each group of patients in Fig. 3b. We find that patients in all groups have an increased number of conditions in post-SMI periods, compared to pre-SMI periods. Compared to younger groups, patients diagnosed with SMI at an older age tend to suffer from a larger number of conditions in both pre- and post-SMI periods. Moreover, we control for repeated observation for chronic diseases, as these diseases can first appear in a patient’s pre-SMI period and then be re-examined or re-treated and re-appear in post-SMI diagnoses, leading to the artificially inflated degrees of patient nodes in the post-SMI network. To this end, we calculate the ratio between the number of conditions that for the first time appear after a SMI diagnosis (i.e., *new conditions*) and the total number of conditions that a patient has. We find that the mean ratio is 0.57, with 95% confidence interval (CI) of (0.56–0.58), suggesting that patients with SMI have more than a half of conditions first appearing after their SMI diagnoses. To verify the robustness of this result, we further examine the mean ratios of new conditions among groups with different first SMI-diagnoses age in Fig. 3c. We find that the high ratios of new conditions in post-SMI periods are widely observed across age groups and most groups have a mean ratio of new conditions higher than 0.5. By inspecting these new conditions across age groups, we find that mental health conditions, e.g., mental disorders due to use of tobacco (“F17”), are more prevalent in groups who were diagnosed with SMI at a younger age, while physical health conditions such as hypertension (“I10”) are more prevalent in groups with an older SMI-diagnosis age (Fig. S6, *SI*).

As people have an older chronological age in post-SMI periods than in pre-SMI periods, this raises another question whether the high incidences of new conditions in post-SMI periods are due to a normative ageing process or related to other complex processes associated with their SMI, such as adverse effects of anti-psychotic medication [58]. To explore this, we examine when these new conditions appear in post-SMI periods. We expect that most new conditions appear in later stages of post-SMI periods if the incidence of a new condition is more related to the normative ageing process. We find that most new conditions in post-SMI periods on average first appear in the first 5 years after an individual was first diagnosed with SMI (Fig. 3d).

#### 3.1.3 Factors associated with multimorbidity progression

**Fig. 4:**
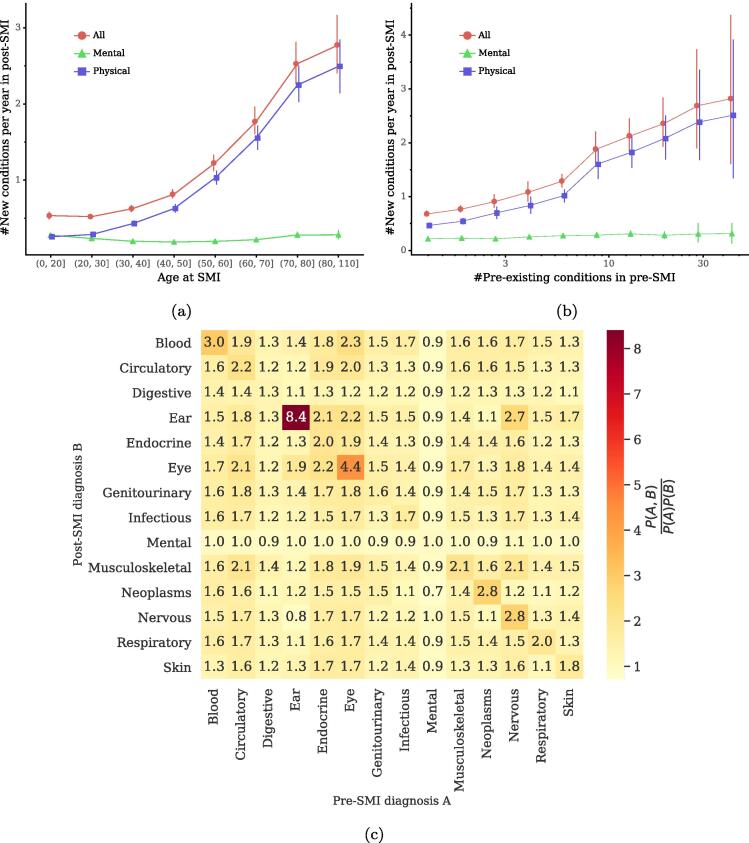
(a) Numbers of new conditions per year in post-SMI periods over age at SMI. (b) Numbers of new conditions per year in post-SMI periods over number of pre-existing conditions in pre-SMI periods. Error bars indicate 95% CIs. (c) The ratio of the observed probability of finding patients with a pre-SMI diagnosis *A* and a post-SMI diagnosis *B* to that expected if the occurrences of *A* and *B* were independent. All diagnoses are grouped by the ICD-10 chapter. A ratio larger than 1 implies that the associations of diagnoses between two chapters are stronger than expected. See *SI* for detailed lists of the prevalent conditions in pre- and post-SMI episodes.

The above results reveal that patients often have a large number of new conditions in post-SMI periods. Analysis on the numbers of patients’ episodes on managing new and re-occurring conditions further shows that managing new diseases is dominating in post-SMI care (Fig. S5, *SI*). We next explore which characteristics of individuals are associated with the number of new diseases in post-SMI periods. However, the absolute numbers of new diseases are not comparable across individuals with different observation periods in our data (Fig. S2e, *SI*); those who are observed over short periods can have less data and display a small number of new conditions in their post-SMI periods. For a fair comparison, here we use a normalized metric which measures the average number of new diseases per year from the date of first SMI diagnosis to March 31, 2018 (the date of administrative censoring) for alive patients and to the date of death for dead patients. We first examine associations between individuals’ demographics and the number of new diseases per year in post-SMI periods. Fig. 4a shows the numbers of new diseases per year in post-SMI periods over the age at SMI diagnoses, in which we also categorize new diseases into mental and physical health conditions to explore differences between types of diseases. We find that most new diseases in post-SMI episodes are physical health conditions, and mental health conditions account for a small proportion across different age groups.

Apart from demographics, we also examine association between individuals’ medical history and the number of new conditions in post-SMI periods. Fig. 4b shows the number of new disease per year in post-SMI periods over the number of pre-existing diseases in pre-SMI periods. We find that patients with more historical conditions tend to have more new diseases in post-SMI periods. Similar to the results in Fig. 4a, most of these new diseases are physical health conditions across different groups. These results together indicate that both individuals’ demographics and their medical history are associated with their health outcomes in post-SMI periods, particularly on the number of new physical health conditions.

To further examine associations between a pre-SMI diagnosis *A* and a post-SMI diagnosis *B*, we calculate the ratio of the observed probability of finding patients with both *A* and *B*, i.e., P(A,B), to that expected by chance, i.e., P(A)P(B). By grouping diagnoses into the ICD-10 chapters, we find that diagnoses in pre-SMI episodes are not strongly associated with a diagnosis of mental health condition in post-SMI episodes, while the associations between pre- and post-SMI diagnoses on two different types of physical health conditions are generally higher than expected (Fig. 4c). This aligns with the above results that the number of pre-existing conditions in pre-SMI episodes is less associated with the number of new mental health conditions in post-SMI episodes but has a strongly positive association with the number of new physical health conditions (Fig. 4b).

### 3.2 Patient-centric analysis

So far, our analysis has been proceeded primarily from a disease-centric perspective, where individuals’ information has been largely aggregated to a group level. Next, we measure connectivity patterns in the patient-diagnosis networks to explore how different individuals connect to different diseases from a more patient-centric perspective.

#### 3.2.1 Interactions between patients and diseases

**Fig. 5:**
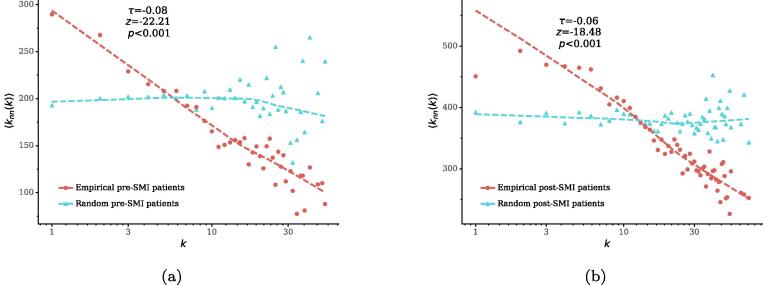
Correlations between node degree *k* and average degree of neighbors $\langle k_{\mathrm{nn}}\rangle$ in the bipartite networks and in their random counterparts. Dotted lines show the LOESS (Locally Estimated Scatterplot Smoothing) curves. The annotation marks the Kendall’s τ coefficient between *k* and $\langle k_{\mathrm{nn}}\rangle$ in the real-world networks, as well as its *z*-score and *p*-value compared to those measured in 1,000 random networks.

We first explore whether nodes tend to connect to other nodes of similar degree, i.e., assortative mixing [59], by measuring degree correlations between patient and disease nodes. To this end, we plot the average degree of direct neighbors of a node $\langle k_{\mathrm{nn}}\rangle$ as a function of its degree *k* in Fig. 5, for patient nodes in pre- and post-SMI network respectively. For comparison, we also plot the same functions obtained from random networks generated by a null model. In both patient and disease nodes, the plots for the random networks are close to horizontal lines, indicating that there are no correlations between a node’s degree and the average degree of its neighbors. In contrast, the plots in the real-world network show negative correlations, i.e., the average degree of neighbors of patient nodes decreases with the nodes’ degree. These results suggest disassortative mixing patterns, i.e., low-degree nodes tend to connect to high-degree nodes. In other words, patients with a smaller number of conditions tend to suffer from prevalent diseases. Similar patterns are observed for disease nodes (*SI*).

#### 3.2.2 Diverse comorbidities in patients affected by prevalent conditions

**Fig. 6:**
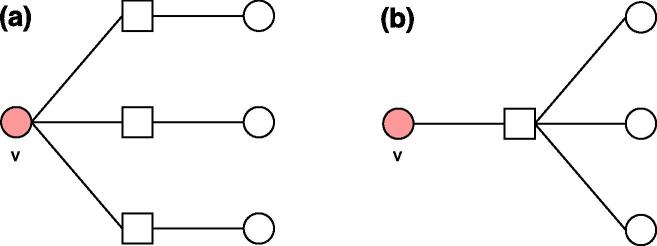
Illustration of correlations between node degree and the number of second-order neighbors, where a circle denotes a disease and a square denotes a patient. Disease node *v* has 3 second-order neighbors in both (a) and (b), but through different processes: (a) *v* affects many patients who have a few other conditions, and (b) *v* affects a single patient who has many other conditions. Different processes can shape different intervention strategies: a group-based intervention is needed if a condition affects a large number of patients, while an individual based intervention should be given if a condition particularly affects certain patients with a specific medical history.

**Fig. 7:**
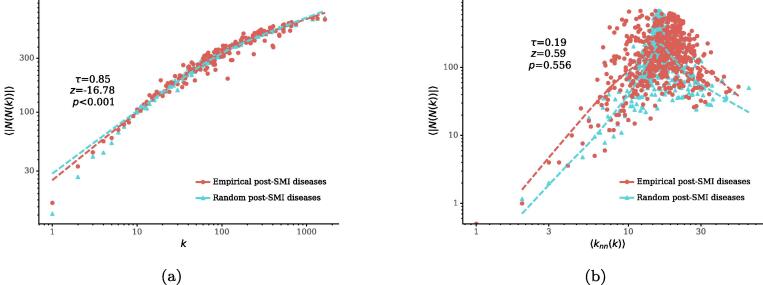
(a) Correlations between node degree *k* and the average number of second-order neighbors 〈|N(N(k))|〉 in the post-SMI network. (b) Correlations between the average degree of neighbors $\langle k_{\mathrm{nn}}(k)\rangle$ and average number of second-order neighbors 〈|N(N(k))|〉 in the post-SMI network. Dotted lines show LOESS curves. The annotation shows the Kendall’s τ coefficient between two variables in the real-world networks, as well as its *z*-score and *p*-value compared to those in 1,000 random networks.

We next extend the correlation analysis from a node’s direct neighbors to its second-order neighbors N(N(v)), i.e., nodes with a distance of 2 from a node *v* but excluding *v*, so as to explore whether a condition has many co-occurrence conditions (i.e., comorbidities) because the condition affects many patients or because it particularly affects patients with many conditions (Fig. 6). If the former explanation applies, we expect a high correlation between the number of a disease node *v*’s second-order neighbors |N(N(v))| and *v*’s degree k(v); otherwise, we expect a high correlation between |N(N(v))| and the average degree of *v*’s direct neighbors $\langle k_{\mathrm{nn}}(v)\rangle$. Fig. 7a plots the average number of second-order neighbors of a disease node 〈|N(N(k))|〉 as a function of its degree *k*. We find that 〈|N(N(k))|〉 has a strongly positive correlation with *k* in the post-SMI network (τ=0.85). Significance tests based on null model show that these correlations are significantly different from random references (p<0.001). In contrast, as shown in Fig. 7b, the correlation between 〈|N(N(k))|〉 and $\langle k_{\mathrm{nn}}\rangle$ is relatively low (τ=0.19) and not significantly different from random one (p=0.556). These results suggest that a large number of co-occurrence conditions that a condition has is likely associated with a large number of patients affected by the condition, i.e., a high prevalence of the condition. This also indicates that individual patients with the same disease tend to have heterogeneous disease processes. Similar results are found in the pre-SMI network (*SI*).

#### 3.2.3 Patients with similar attributes connect to the same diseases

**Fig. 8:**
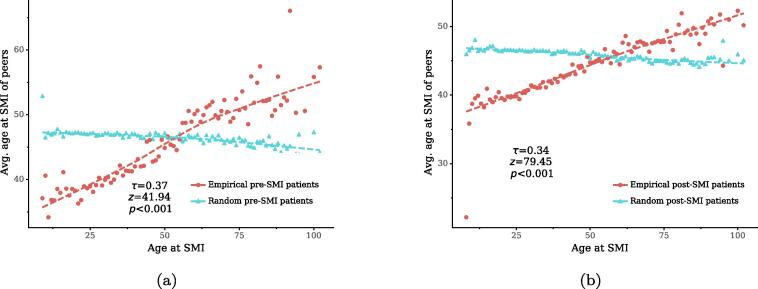
Correlations of age at SIM with peers’ age at SMI in the bipartite networks and in their random counterparts. Dotted lines show LOESS curves. The annotation shows the Kendall’s τ coefficient between two variables in the real-world networks, as well as its *z*-score and *p*-value compared to those in 1,000 random networks.

In a similar way, we examine correlations of characteristics between a patient node and its second-order neighbors to explore whether patients with similar characteristics tend to have the same conditions. Fig. 8 plots the average age at SMI diagnosis of a patient node’s second-order neighbors as a function of the node’s SMI-diagnosis age. The average age at SMI diagnosis of second-order neighbors of a patient node grows almost linearly with the age at SMI diagnosis of the node in both pre-SMI and post-SMI periods. These positive correlations are significantly higher than expected by chance (p<0.001), suggesting that patients diagnosed with SMI at a similar age tend to suffer from the same conditions. Moreover, by measuring modularity [60] in the projected one-mode networks, we find that patients with the same gender and ethnicity tend to suffer from the same conditions as well (see Table S6, *SI*). These results suggest that patient nodes with similar attributes tend to cluster by connecting to the same disease nodes.

### 3.3 Analysis of network evolution

The above results show that patient and disease nodes with similar attributes tend to cluster in the bipartite networks. However, it is unclear when nodes start to cluster and which process (e.g., disease progression or other mechanisms) drive the clustering of nodes. Next, we explore these in detail by exploiting the high temporal resolution in diagnosis data.

#### 3.3.1 Trend of distances

**Fig. 9:**
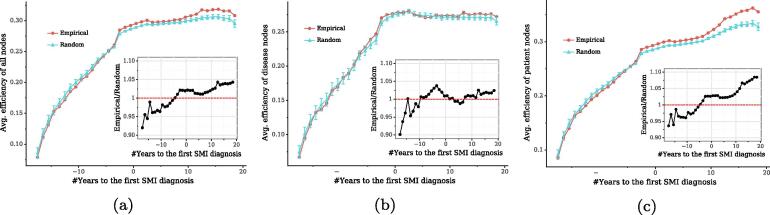
Average efficiencies of nodes in bipartite networks and in their random counterparts over time, where a sliding window of 5 years is used here. Error bars show 95% CIs of the average efficiencies in random networks and the x-axes show the mean value of two boundaries of a sliding window, i.e., $t_{i}+\Delta t/2$. Each inset shows the ratios between the empirical and random average efficiencies over time, and the red horizontal line marks the point where the empirical average efficiency is equal to random one. For each temporal bipartite network, 1,000 random networks are generated by randomly shuffling connections between patient and disease nodes while preserving their degree sequences.

We first examine nodes’ clustering trends by measuring their distances in the bipartite networks over time. We build temporal bipartite networks based on patients’ episodes within sliding time windows and measure nodes’ distances of these networks by the average efficiency [45]. Fig. 9a shows the average efficiencies of nodes in the temporal bipartite networks, in which a sliding window of 5-year length is used. As shown by the red line in Fig. 9a, the average efficiencies of nodes increase over time, although the increase is less substantial after the first diagnoses of SMI. This indicates that overall the distances of nodes are decreasing and nodes are integrated over time.

As patients may have more hospital visits at an older age and existing conditions that often require treatment and monitoring tend to be recorded in recent episodes, one might wonder whether a larger number of diagnoses at a later stage account for the decreased distances. One approach to answer this is to compare the empirical trend with the efficiencies in random networks that have the same degrees as the original networks, i.e., control for the number of diagnoses that a patient had at each stage. The blue line in Fig. 9a shows the average efficiencies in random networks, which serve as references. The ratio between the empirical efficiencies and the reference values is increasing, which suggests that more complex processes, rather than more diagnoses recorded at a later stage alone, are responsible for the decreased distances in the patient-diagnosis networks.

#### 3.3.2 Processes driving evolution

**Fig. 10:**
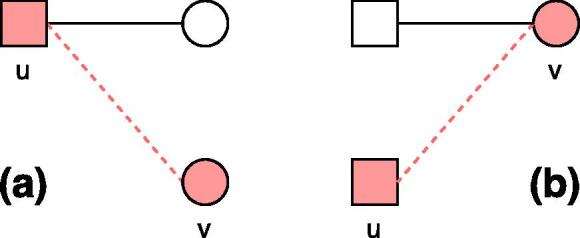
Clustering of nodes in bipartite networks, where a square denotes a patient and a circle denotes a disease. Adding a link between *u* and *v* results in the decreased distances of nodes in both (a) and (b), but through two distinct processes: a patient who has existing conditions tends to suffer from a new condition in (a), while a new patient tends to suffer from the same condition as other patients in (b). Although the two different clustering processes decrease the same amount of overall distances of all nodes in the networks, the distance of disease nodes (i.e., circles) decreases more substantially in (a) and the distance of patient nodes (i.e., squares) decreases more substantially in (b).

One might wonder whether the complex processes leading to the decreased distances are (a) disease progression [30], [28], [31], [11], i.e., a patient with some existing conditions tends to suffer from another condition, or (b) selection effects [61], i.e., some patients tend to be diagnosed with the same conditions due to common characteristics such as demographic and genetic attributes. To approach this, we examine the efficiencies of patient nodes and disease nodes respectively. We expect that the efficiencies of disease nodes are much higher than expected by chance if disease progression explains the decreased distances, while we expect that the efficiencies of patient nodes are higher if selection effects matter (see Fig. 10).

We find that the empirical values of efficiencies among disease nodes are close to those expected in random networks (Fig. 9b). In contrast, the average efficiencies of patient nodes in the real-world networks are rising over time and increasingly higher than random ones (Fig. 9c). These results indicate that patients in our cohort tend to be diagnosed with the same conditions, showing a stronger contribution than disease progression to the shortened distances of nodes in the patient-diagnosis networks. This aligns with our finding that patients with similar attributes tend to suffer from the same conditions in Fig. 8. Similar results were observed when using different lengths of sliding windows, e.g., 10 years. Moreover, to control for the fact that some conditions may have long-term impacts on people’s wellbeing and lead to other comorbidities after years, beyond the time windows used above, we repeated the above analyses based on cumulative temporal networks, i.e., a temporal network at time $t_{i}$ aggregates all episodes that were recorded at $t<=t_{i}$. Again, the results were not altered.

#### 3.3.3 Dynamic graphlets

**Fig. 11:**
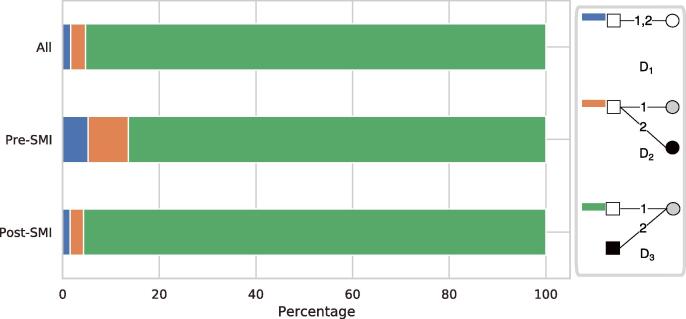
Percentages of all 2-edge temporal graphlets within a time window of 5 years, in all, pre- and post-SMI episodes respectively.

To further capture the temporal ordering of edges and better understand the clustering processes, we examine graphlets in temporal bipartite networks. We expect that graphlet $D_{2}$ is more frequent than $D_{3}$ if the clustering in the patient-diagnosis network is more driven by disease progress, while $D_{3}$ is more frequent if the clustering is driven by selection effects (Fig. 2b). Fig. 11 shows the fractions of all 2-edge temporal graphlets in a 5-year window. We find that $D_{3}$ is the most dominating graphlet in all episodes, with 337 M occurrences accounting for 95.3% of all 2-edge temporal graphlets, much more than $D_{1}$ and $D_{2}$. As all patients in our cohort had a SMI diagnosis, it may not be surprising that $D_{3}$ is common in the temporal graphlets. To eliminate the effect of data selection, we also examine the temporal graphlets in pre-SMI episodes which do not contain a SMI diagnosis. We find that $D_{3}$ accounts for the majority of temporal graphlets (86.4%) in pre-SMI episodes as well, although the fractions of D1 and $D_{2}$ are relatively higher than those in all episodes and post-SMI episodes (Fig. 11). Robustness checks with different lengths of a time window (e.g., 1 year) produced similar results. These results confirm our previous results in the distance analysis, i.e., the clustering of patients and diseases is largely driven by the fact that patients with similar attributes tend to be diagnosed with the same conditions.

## 4 Discussion

In this paper, we have systematically examined multimorbidity patterns among patients with SMI (schizophrenia and bipolar disorder) based on a large set of linked patient data that comprises comprehensive SMI diagnoses of patients in South London and longitudinal data on their admissions to NHS hospitals, By representing patients’ diagnoses in admissions using temporal bipartite network models, we have characterized multimorbidity profiles of patients with SMI, measured changes of multimorbidity before and after their first SMI diagnoses, identified associations between these changes and patients’ characteristics, and quantified interactions between patients and diseases. Moreover, our analysis on the evolution of network structures over time sheds light on the multimorbidity processes among these patients over their illness duration of SMI.

While comparisons to other studies are difficult due to heterogeneity in study designs, condition definitions and populations [62], our results align with previous cross-sectional studies on multimorbidity among patients with SMI [5], [14], [21]. We find that 63.7% of patients in our cohort have more than 2 different health conditions (including SMI) in secondary care after their first SMI diagnoses. This aligns with previous studies where multimorbidity was found to be prevalent among people with SMI in primary care [18], [4]. Among comorbidities of patients with SMI, we find that the most common physical health conditions are endocrine, respiratory, digestive and circulatory diseases. This is highly consistent with a Danish nationwide cohort study where respiratory, digestive, and cardiovascular diseases were found to have the strongest associations with mortality among patients with schizophrenia [5], and multiple meta analyses on physical comorbidities across patients with mental disorders [16]. Also, our results show that tobacco use or smoking is one of the most common unhealthy lifestyle behaviors after patients experience SMI, particularly among younger groups. Similar findings have been reported in other studies [15], [14], [16]. Moreover, we find that older patients tend to suffer from a larger number of conditions, in line with prior evidence that the prevalence of multimorbidity generally increases with age [20], [7], [21].

Although recent studies have exploited longitudinal data to understand multimorbidity among people with SMI, these studies have largely focused on measuring an overall trend in the prevalence of multimorbidity over chronological time or age rather than illness duration of SMI [18]. This study presents a complementary analysis that explores developmental trajectories of multimorbidity over the illness duration of SMI, which reveals several new findings. First, we find that on average 57% of conditions affecting patients with SMI first appear after being diagnosed with SMI. These new conditions often appear in the first years after a SMI diagnosis, suggesting that the normative ageing process alone is not responsible for the high incidences of new conditions in post-SMI periods. Further research is needed to understand whether the relationship between SMI and new conditions is because SMI contributes to the development of these conditions, or because more frequent hospital visits for treatment or examination in post-SMI periods lead to a better documentation of these conditions [3], [18]. Second, we find that patients who are diagnosed with SMI at an older age and those who have a larger number of pre-existing conditions in pre-SMI periods tend to suffer from a larger number of new conditions in post-SMI periods, particularly on physical health conditions. This confirms the importance of both demographic characteristics and medical history in understanding the development of human diseases [30]. Finally, previous studies show that mental health conditions, such as depression and anxiety, are often associated with the occurrences of hearing impairment and arthritis [63], [29]. In our association analysis between pre- and post-SMI diagnoses, we have not found a strong association between mental illnesses and ear or musculoskeletal conditions. However, we find that patients who experienced nervous conditions at an early stage tend to suffer from ear, musculoskeletal and mental health conditions at a later stage. This implies that nervous conditions may act as a confounding factor that influences the incidences of both mental illnesses and ear, musculoskeletal illnesses, leading to an association among these illnesses.

Unlike previous network-modeling based studies that often compress patient-diagnosis data in a traditional one-mode network [33], [29], [30], [28], [31], [11], which often results in information loss in data representation [64], [54], we here use bipartite networks to jointly model patients’ demographic characteristics and medical histories. These bipartite network models provide a natural representation of relationships between patients and diseases in diagnosis data and avoid information loss, which allows us to examine the interactions between individual patients’ attributes and disease progression paths, extending previous studies on network medicine from the population level to an individual level. Based on these models, this study has revealed for the first time the relational structure between patients and diseases, going beyond the relational structure among a single set of nodes, such as diseases’ co-occurrence relations in existing studies [33], [29], [30]. We find that the bipartite networks show disassortative mixing by degree, i.e., patients with a small number of conditions tend to connect to prevalent diseases. This result can be relevant to public health, e.g., prevention strategies that target prevalent conditions may strongly destroy the connections in these bipartite networks and improve patients’ well-being, particularly for those affected by a small number of conditions. Prevention for prevalent diseases becomes even more vital, when considering another finding that prevalent conditions tend to have a wide range of co-occurrence conditions. It is well known that individuals with multiple conditions have complex care needs, and they are often the most costly and challenging patients to manage [7]. Thus, effective prevention or intervention programs for prevalent diseases may help to reduce combinations of multiple conditions in a single individual and improve care outcomes. We also find that patients with similar attributes, such as demographics, tend to experience the same conditions, which provides practical tips for detecting individuals at risk of these prevalent diseases, i.e., identifying those who have similar attributes as existing patients with a prevalent disease, which aligns with existing methodologies of diagnostic prediction models [65], [66], [67].

Furthermore, our analyses on the temporal evolution of patient-diagnosis networks reveal that patients and diseases become more interconnected over time, along with the illness duration of SMI. This clustering trend is more pronounced in post-SMI periods, as compared with null models. Our results also suggest that the clustering process is more likely to be driven by the fact that patients with similar attributes tend to suffer from the same conditions than disease progression. This is not surprising, as a rich body of research has shown that the onsets of both physical health conditions, e.g., cardiovascular diseases [68], [66] and diabetes [65], and mental health conditions such as psychosis [67], [69], are associated with a range of risk factors such as demographics, socioeconomic status, lifestyle behaviors (e.g., smoking and alcohol intake) and genetic attributes captured by family history, rather than disease progression alone. However, our results differ from prior network-based studies on disease trajectories, in which diseases progression is deemed as a main factor driving the clustering of diseases [30], [28], [31], [11]. One reason for this difference is that prior studies have largely focused on modeling relationships among diseases/symptoms at a population level by using one-mode network models, without considering the relationships between individual patients and diseases. In other words, these models assume homogeneity in patients and neglect disease heterogeneity, i.e., each patient may develop a distinct pathway in disease development [34], [70]. Ignoring such heterogeneity in patients – by looking only at aggregates across a whole cohort – can lead to an overestimate of disease progression. For example, if a group of patients are often diagnosed with disease *i* followed by a subsequent diagnosis of disease *j* and another group of patients are diagnosed with disease *j* followed by a subsequent diagnosis of disease *k*, a progression path between *i* to *k*, i.e., i→j→k, is derived in these one-mode networks models. This can be problematic, as the two progression paths i→j and j→k are identified from two different groups respectively and may not be transitive among the same group of patients, i.e., i→j→k. Thus, ignoring heterogeneity in patients can result in misleading results on disease progression. In contrast, our temporal bipartite models take information on both patients and diseases into account, which allows us to distinguish the clustering patterns of diseases driven by disease progression and those driven by other factors, with control for patient heterogeneity.

Our work has its limitations. First, due to the restrictions of data linkage policies, we only have access to HES data for patients who had used a service at SLaM and do not have data for patients without a mental health condition. As a result, there is a lack of control/reference groups in our study, which makes it hard to explore whether and how a diagnosis of SMI influences patients’ diagnoses on other diseases when compared to the general population. Second, although our dataset covers 22 years of inpatient data for SMI patients within South London, we only included diagnoses generated in inpatient admissions. We did not include diagnoses in outpatient appointments, and accident and emergency visits in HES data, because (1) the focus of this study is to investigate patients’ hospitalization which involves the most costly healthcare services [71] and (2) inpatient data have been recorded for a longer time and have higher quality in data recording [72]. Also, milder diseases and symptoms that are often diagnosed and treated by general practitioners might not be included in our data. Third, since HES data were originally constructed for billing purposes, some diseases may be over-recorded or mis-recorded to meet reimbursement criteria. Also, while we searched both HES and CRIS data to identify an accurate date that a patient was first diagnosed with SMI, there may be a delay from the date that a patient first experienced symptoms to the date recorded in EHR data. Similar issues may exist for the diagnosis dates of other conditions. Finally, in this work, we have focused on examining an overview of temporal multimorbidity profiles for the whole SMI (focusing on schizophrenia and bipolar disorder) cohort. Future research is needed to examine the differences between groups affected by different types of SMI. However, given a high mis-diagnostic ratio between some types of SMI [57], special attention is needed in identifying the cohort with a specific type of SMI to avoid misleading results in these comparison studies.

## 5 Conclusion

This paper presents an approach based on temporal bipartite network models to characterize hospitalization patterns and multimorbidity profiles for patients with SMI. The proposed models provide a natural, flexible and unified framework to integrate and represent rich information in patient-diagnosis data, which allows a deeper understanding of relationships between patient attributes and diseases, going beyond the associations of diseases studied by using traditional one-mode network models. We find that the resulting bipartite networks display disassortative mixing patterns, i.e., patients with a small number of conditions tend to connect to more prevalent diseases, and both demographics and medical history strongly determine the network structures. Our analysis on the temporal evolution of these networks further reveals that patient and disease nodes become more interconnected over the illness duration of SMI, mainly driven by the process that patients with similar attributes tend to be diagnosed with the same conditions. Our results reveal developmental trajectories of multimorbidity over the illness duration of SMI, which can help healthcare professionals decide on treatment and prevention for patients with SMI and improve patient well-being.

Our work also provides a basis to further investigate temporal trajectories of multimorbidity by jointly modeling multiple risk factors of individuals such as demographic, clinical, socioeconomic and environmental factors. Future research on applying network embedding methods which map nodes in temporal bipartite networks to vectors of real numbers in a multidimensional space [73], [74] can offer further insights into the course of a disease and the relationships between diseases, as the resulting vector representations can be used for various network-based disease analysis tasks such as link prediction (i.e., determining the risk of a disease for an individual), detecting clusters of diseases, predicting an attribute of a patient/disease node and removing noise information (e.g., mis-diagnoses) in raw data.

## Declaration of Competing Interest

The authors declare that they have no known competing financial interests or personal relationships that could have appeared to influence the work reported in this paper.

## Footnotes

1 Raw pre-SMI and post-SMI networks have highly distinct structures by definition, because there is no disease node on SMI in a pre-SMI network, while each patient node links a SMI node in post-SMI networks, leading to SMI nodes to be super hubs in post-SMI networks. This inherent difference makes pre- and post-SMI networks incomparable.
