# Supplementary material for: Patient-centric characterization of multimorbidity trajectories in patients with severe mental illnesses: A temporal bipartite network modeling approach
Source: J Biomed Inform. 2022 Mar;127:104010. doi: 10.1016/j.jbi.2022.104010 (PMC8894882; doi:10.1016/j.jbi.2022.104010)
Supplement: Supplementary data 1 [file mmc1.pdf]

Supplemental Information for: Patient-centric characterization of multimorbidity trajectories in patients with severe mental illnesses: A temporal bipartite network modeling approach

Tao Wang, Rebecca Bendayan, Yamiko Msosa, Megan Pritchard, Angus Roberts, Robert Stewart, and Richard Dobson

Contents

1 Descriptive statistics **2**

2 Prevalent diseases **4**

3 Post-SMI care patterns **8**

4 Network structures **9**

List of Figures

S1 Distribution of age at first SMI diagnosis. 3
S2 Statistics of HES episodes 3
S3 Degree distributions 4
S4 Average degrees of disease nodes by the ICD-10 categories/chapters. 5
S5 Care focus on new and re-occurring conditions in post-SMI episodes 8
S6 The top 10 prevalent new and re-occurring diseases per SMI-diagnosis age group . 10
S7 Correlations between node degree and average degree of neighbors 11
S8 Correlations between node degree and the average number of second-order neighbors 11

List of Tables

S1 Demographic characteristics of patients 2
S2 Top 10 prevalent conditions in pre-SMI periods 5
S3 Top 10 prevalent conditions in post-SMI periods 5
S4 The most connected disease nodes with patients with schizophrenia 6
S5 The most connected disease nodes in patients with bipolar affective disorder 7
S6 Modularity by attribute in projected one-mode networks of patient nodes 9

1 Descriptive statistics

We first present descriptive statistics of patients' demographics. Table S1 summarizes demographic characteristics of patients, grouped by their first SMI diagnoses. Among all 7,728 patients, 4,636 (60%) patients were first diagnosed with schizophrenia ("F20") and the rest 3,092 patients were first diagnosed with bipolar ("F31"). We compare characteristics between groups of patients by using t-tests for continuous variables and χ^2 tests for categorical variables. We find that the mean age at the first diagnoses of "F20" is 39.5 with a standard deviation (SD) of 16.7, and the mean age at the first "F31" diagnoses is 39.4 (SD=16). No significant difference was found between two groups in these age distributions ($P = 0.88$). Figure S1 shows the distribution of patients' age at their first SMI diagnoses. However, two groups show significantly different distributions in gender and ethnicity ($P < 0.001$). Specifically, the majority of "F20" patients (61.4%) were male, while "F31" patients were dominated by females (62.8%). A notable result in the comparisons of ethnicity is that the fraction of black patients is much higher in the "F20" group (37.3%) than that in the "F31" group (16.4%). These results highlight the relevance of demographic features in disease development.

Table S1: Demographic characteristics of patients, stratified by the first SMI diagnoses. P-values for two-tailed tests with the Bonferroni correction, where * $p < 0.05/m$, ** $p < 0.01/m$, *** $p < 0.001/m$, and $m = 3$.

Characteristic	F20 (N=4,636)	F31 (N=3,092)	All (N=7,728)	P
**Age at diagnosis**				0.88
Mean	39.5	39.4	39.5	
SD	16.7	16	16.4	
Range	[9, 102]	[8, 95]	[8, 102]	
**Gender**				<0.001***
Female	1,788 (38.6%)	1,936 (62.6%)	3,724 (48.2%)	
Male	2,848 (61.4%)	1,156 (37.4%)	4,004 (51.8%)	
**Ethnicity**				<0.001***
White	2,010 (43.4%)	1,916 (62%)	3,926 (50.8%)	
Black	1,724 (37.2%)	510 (16.5%)	2,234 (28.9%)	
Asian	317 (6.8%)	166 (5.4%)	483 (6.3%)	
Mixed	120 (2.6%)	77 (2.5%)	197 (2.5%)	
Other	295 (6.4%)	251 (8.1%)	546 (7.1%)	
Missing	170 (3.7%)	172 (5.6%)	342 (4.4%)	

We next present descriptive statistics of HES records. Figures S2a and S2b show the numbers of episodes and patients in HES data per year. Although the data are not equally distributed across years and the majority of data are collected between 2008 to 2018, the amount of data in each year is sufficient to draw meaningful conclusions. We further examine how these episodes are distributed across patients. We count the numbers of episodes that each patient has in our dataset and show the distribution of these counts in Figure S2c. We find that a large number of patients have a relatively small number of episodes (e.g., 50% of patients have less than 7 episodes) and a small number of patients have a large number of episodes with the maximum number of 2,026, following a long tail distribution. Also, we examine the duration of each episode by counting the number of days from admission to discharge, where 1 day is added in all periods to include episodes where patients were admitted and discharged within the same day. As shown in Figure S2d, the duration lengths of episodes also display a long-tail distribution, where most episodes have a short period with a mean of 21.9 days (SD=86.3) and a small number of episodes have an extremely long period with the longest period of 3,435 days. By counting the number

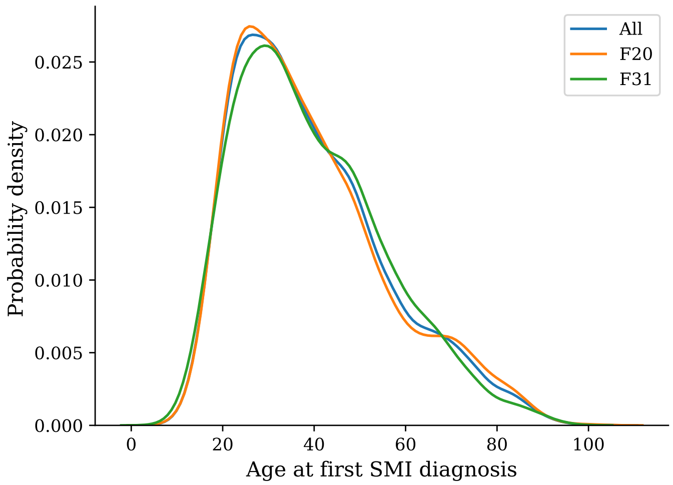

Figure S1: Distribution of age at first SMI diagnosis.

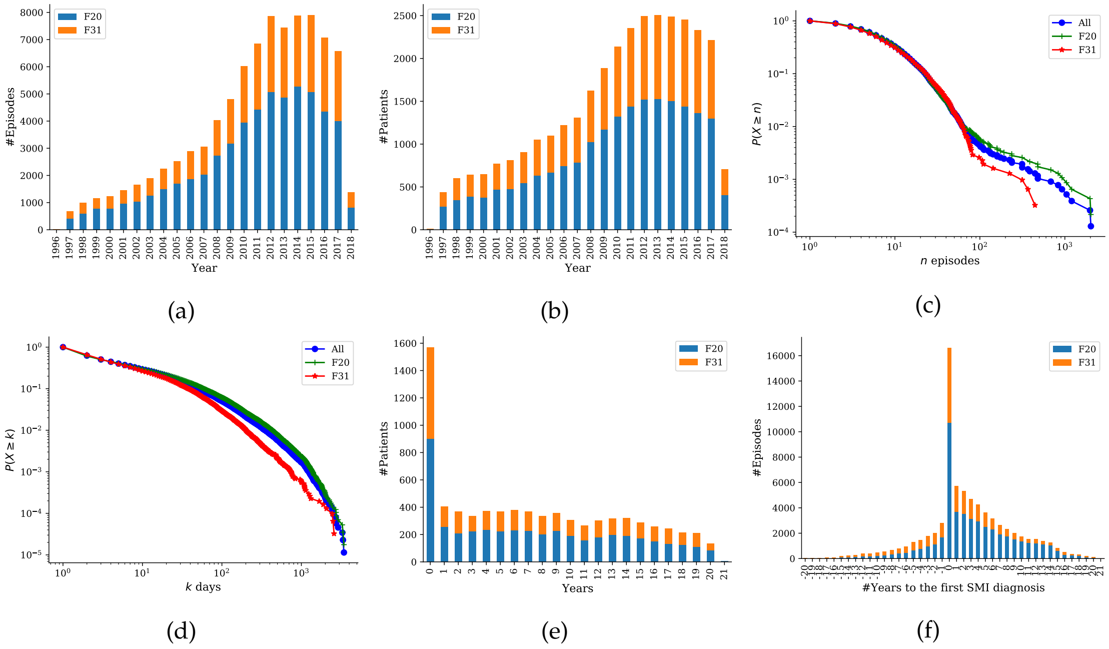

Figure S2: Statistics of HES episodes. (a) Numbers of episodes per year. (b) Numbers of patients per year. (c) Distribution of episode counts per patient. (d) Distribution of duration of each episode. (e) Numbers of patients over care lengths. (f) Numbers of episodes before and after first SMI diagnoses.

of years from the admission date of a patient's first episode to the discharge date of her/his last episode in our data, we further examine the length of care history of each patient covered in our data. As shown in Figure S2e, the lengths of these periods range from 0 to 21 years, with the average length of 8.3 years and SD=6.3. Another pattern in this figure is that the numbers of patients with different care lengths are much comparable, except those less than 1 year, implying that our samples are representative across patients with various intensity levels of care service use. Moreover, we calculate the numbers of episodes before and after a patient was diagnosed with SMI. The numbers of episodes over years to the first SMI diagnoses are shown in Figure S2f, which clearly shows a pattern that patients have more episodes after a SMI diagnosis.

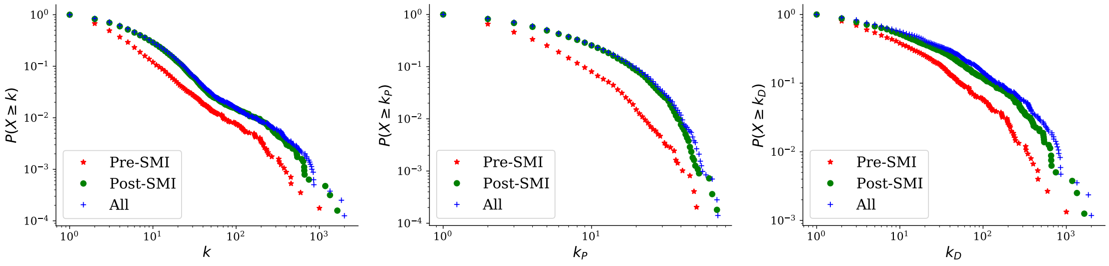

(a) (b) (c)

Figure S3: Degree distributions in different networks. (a) Degree distributions of all nodes. (b) Degree distributions of patient nodes. (c) Degree distributions of disease nodes.

Figures S3 shows the degree distributions of nodes in networks built on pre-SMI, post-SMI and all epsiodes respectively. All distributions display a long tail, meaning that most nodes have a low degree and a small number of nodes have a extremely high degree.

2 Prevalent diseases

By examining degree of each disease node in networks, we show the most 10 prevalent conditions in pre- and post-SMI periods in Tables S2 and S3 respectively. For each condition, we calculate the number of patients affected by the condition and its prevalence rates among patients in our constructed networks. As mentioned in the main text, a patient who only had a SMI diagnosis and did not have diagnoses on other diseases were not included in the network models, and hence the number of patients in networks is smaller than that in the whole cohort. We find that mental health conditions dominate the most prevalent conditions in both pre- and post-SMI periods, such as depressive episode ("F32") in pre-SMI periods and mental disorders due to use of tobacco ("F17") in post-SMI periods. However, compared to pre-SMI periods, there are a larger number of physical health conditions commonly appearing in post-SMI periods. Moreover, endocrine, nutritional and metabolic diseases such as "E11" and "E78" are more prevalent in post-SMI periods than in pre-SMI periods.

We also show the most common health conditions among patients, stratified based on their first SMI diagnoses: schizophrenia (F20) and bipolar affective disorder (F31) in Tables S4 and S5 respectively. Here, we only include conditions with a prevalence rate higher than 5%. To estimate the timing order of each condition's incidence, we compute the average number of years from the date of a patient's first diagnosis of a condition to the date of the patient's first diagnosis of SMI, as well as their standard deviations.

To explore difference of pre- and post-SMI multimorbidity profiles among patients with different SMIs, we stratify patients based on their first SMI diagnoses (i.e., schizophrenia or bipolar affective disorder) and build temporal bipartite networks for the two different groups respectively.

Table S2: Top 10 prevalent conditions in pre-SMI periods.

Code	Disease name	Chapter	#Patients	%Patients
F32	Depressive episode	Mental	1,002	14.1%
F23	Acute and transient psychotic disorders	Mental	595	8.4%
I10	Essential (primary) hypertension	Circulatory	457	6.4%
F29	Unspecified nonorganic psychosis	Mental	454	6.4%
J45	Asthma	Respiratory	400	5.6%
F99	Mental disorder, not otherwise specified	Mental	376	5.3%
F10	Mental and behavioural disorders due to use of alcohol	Mental	345	4.8%
F17	Mental and behavioural disorders due to use of tobacco	Mental	328	4.6%
K02	Dental caries	Digestive	325	4.6%
N39	Other disorders of urinary system	Genitourinary	268	3.8%

Table S3: Top 10 prevalent conditions in post-SMI periods.

Code	Disease name	Chapter	#Patients	%Patients
F17	Mental and behavioural disorders due to use of tobacco	Mental	1,645	23.1%
F32	Depressive episode	Mental	1,343	18.9%
I10	Essential (primary) hypertension	Circulatory	1,179	16.6%
E11	Non-insulin-dependent diabetes mellitus	Endocrine	749	10.5%
N39	Other disorders of urinary system	Genitourinary	665	9.3%
E78	Disorders of lipoprotein metabolism and other lipidaemias	Endocrine	659	9.3%
J45	Asthma	Respiratory	657	9.2%
F10	Mental and behavioural disorders due to use of alcohol	Mental	640	9.0%
F25	Schizoaffective disorders	Mental	640	9.0%
J18	Pneumonia, organism unspecified	Respiratory	615	8.6%

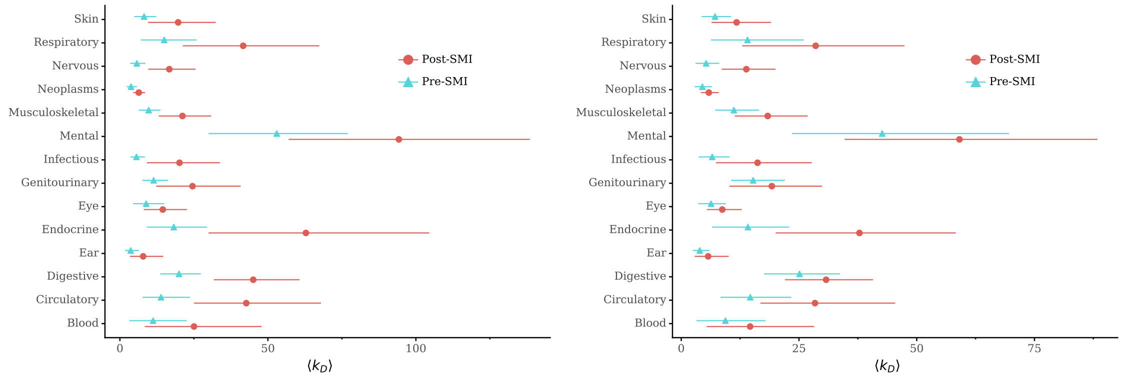

(a) (b)

Figure S4: Average degrees of disease nodes by the ICD-10 categories/chapters among patients with (a) schizophrenia and (b) bipolar affective disorder respectively. Error bars in all plots indicate 95% confidence intervals (CIs).

We then repeat the analysis in the main text and examine degrees of disease nodes by the ICD-10 chapters. Figure S4 shows average degrees of disease nodes in pre- and post-SMI networks by the ICD-10 categories among schizophrenia and bipolar groups. In general, the average degrees of disease nodes in the schizophrenia group are higher than those in the bipolar group, partly because of a larger sample size in the schizophrenia group. Compared to the bipolar group, endocrine, digestive and circulatory diseases in the schizophrenia group have a significantly higher degree in post-SMI networks than in pre-SMI networks, i.e., highly increased incidences of these

Table S4: The most connected disease nodes with patients who were first diagnosed with schizophrenia (N=4,190), ordered by $\langle \Delta t(SMI) \rangle$ the average number of years from the date of the first diagnosis of a condition to the date of the first diagnosis of schizophrenia. A negative value of $\langle \Delta t(SMI) \rangle$ indicates that a condition in general occur before a diagnosis of SMI, and $\sigma(\Delta t(SMI))$ denotes the standard deviation of the time intervals.

ICD-10 Code	Disease name	ICD-10 chapter	Patient counts	Prevalence	$\langle \Delta t(SMI) \rangle$	$\sigma(\Delta t(SMI))$
F23	Acute and transient psychotic disorders	Mental	585	13.96%	-1.8	4.18
F22	Persistent delusional disorders	Mental	356	8.50%	0.1	5.53
K02	Dental caries	Digestive	365	8.71%	0.7	7.13
F29	Unspecified nonorganic psychosis	Mental	653	15.58%	0.7	4.34
F32	Depressive episode	Mental	992	23.68%	0.8	6.13
F99	Mental disorder, not otherwise specified	Mental	549	13.10%	1.0	5.24
J45	Asthma	Respiratory	433	10.33%	1.2	7.29
F10	Mental and behavioural disorders due to use of alcohol	Mental	519	12.39%	1.3	6.26
K29	Gastritis and duodenitis	Digestive	300	7.16%	1.4	7.34
F60	Specific personality disorders	Mental	394	9.40%	1.6	5.79
F12	Mental and behavioural disorders due to use of cannabinoids	Mental	400	9.55%	1.6	5.46
F19	Mental and behavioural disorders due to multiple drug use and use of other psychoactive substances	Mental	394	9.40%	1.7	6.27
M54	Dorsalgia	Musculoskeletal	213	5.08%	1.7	6.59
K44	Diaphragmatic hernia	Digestive	221	5.27%	1.9	7.63
I25	Chronic ischaemic heart disease	Circulatory	223	5.32%	2.3	6.77
D50	Iron deficiency anaemia	Blood	230	5.49%	2.5	7.08
I10	Essential (primary) hypertension	Circulatory	828	19.76%	2.5	6.57
D64	Other anaemias	Blood	329	7.85%	2.7	6.74
F25	Schizoaffective disorders	Mental	518	12.36%	2.9	5.68
G40	Epilepsy	Nervous	216	5.16%	2.9	6.92
N39	Other disorders of urinary system	Genitourinary	467	11.15%	3.0	6.23
F41	Other anxiety disorders	Mental	336	8.02%	3.1	6.09
L03	Cellulitis	Skin	221	5.27%	3.2	6.56
J22	Unspecified acute lower respiratory infection	Respiratory	304	7.26%	3.3	6.84
E11	Non-insulin-dependent diabetes mellitus	Endocrine	570	13.60%	3.4	6.67
K92	Other diseases of digestive system	Digestive	212	5.06%	3.5	6.40
E86	Volume depletion	Endocrine	291	6.95%	3.8	5.25
B96	Other bacterial agents as the cause of diseases classified to other chapters	Infectious	277	6.61%	3.8	5.78
K59	Other functional intestinal disorders	Digestive	389	9.28%	4.0	6.28
E78	Disorders of lipoprotein metabolism and other lipidaemias	Endocrine	468	11.17%	4.1	6.12
J18	Pneumonia, organism unspecified	Respiratory	458	10.93%	4.5	6.37
N17	Acute renal failure	Genitourinary	368	8.78%	4.9	5.34
F31	Bipolar affective disorder	Mental	243	5.80%	4.9	4.51
E87	Other disorders of fluid, electrolyte and acid-base balance	Endocrine	374	8.93%	5.1	5.73
E66	Obesity	Endocrine	277	6.61%	5.1	6.10
F17	Mental and behavioural disorders due to use of tobacco	Mental	1,137	27.14%	5.4	5.67
J44	Other chronic obstructive pulmonary disease	Respiratory	218	5.20%	5.8	6.59

Table S5: The most connected disease nodes with patients who were first diagnosed with bipolar affective disorder (N=2,926), ordered by $\langle \Delta t(SMI) \rangle$ the average number of years from the date of the first diagnosis of a condition to the date of the first diagnosis of schizophrenia. A negative value of $\langle \Delta t(SMI) \rangle$ indicates that a condition in general occur before a diagnosis of SMI, and $\sigma(\Delta t(SMI))$ denotes the standard deviation of the time intervals.

ICD-10 Code	Disease name	ICD-10 chapter	Patient counts	Prevalence	$\langle \Delta t(SMI) \rangle$	$\sigma(\Delta t(SMI))$
K02	Dental caries	Digestive	246	8.41%	-2.7	6.41
K52	Other noninfective gastroenteritis and colitis	Digestive	193	6.60%	-2.5	7.05
F33	Recurrent depressive disorder	Mental	238	8.13%	-1.5	5.65
F23	Acute and transient psychotic disorders	Mental	195	6.66%	-1.5	5.02
J45	Asthma	Respiratory	420	14.35%	-1.5	6.81
F32	Depressive episode	Mental	1,009	34.48%	-0.8	5.48
K62	Other diseases of anus and rectum	Digestive	171	5.84%	-0.8	6.51
F30	Manic episode	Mental	291	9.95%	-0.4	4.31
K29	Gastritis and duodenitis	Digestive	227	7.76%	-0.4	6.53
K44	Diaphragmatic hernia	Digestive	173	5.91%	-0.1	6.76
F10	Mental and behavioural disorders due to use of alcohol	Mental	325	11.11%	-0.1	6.33
M54	Dorsalgia	Musculoskeletal	244	8.34%	0.0	6.70
F29	Unspecified nonorganic psychosis	Mental	207	7.07%	0.4	5.05
F12	Mental and behavioural disorders due to use of cannabinoids	Mental	164	5.60%	0.4	4.93
F60	Specific personality disorders	Mental	277	9.47%	0.5	5.75
F99	Mental disorder, not otherwise specified	Mental	256	8.75%	0.9	5.36
I25	Chronic ischaemic heart disease	Circulatory	155	5.30%	1.0	6.93
F41	Other anxiety disorders	Mental	371	12.68%	1.2	5.02
N39	Other disorders of urinary system	Genitourinary	377	12.88%	1.3	6.72
I10	Essential (primary) hypertension	Circulatory	515	17.60%	1.3	6.42
J22	Unspecified acute lower respiratory infection	Respiratory	185	6.32%	2.0	6.50
M79	Other soft tissue disorders, not elsewhere classified	Musculoskeletal	173	5.91%	2.0	6.69
E11	Non-insulin-dependent diabetes mellitus	Endocrine	243	8.30%	2.0	6.70
D64	Other anaemias	Blood	208	7.11%	2.0	6.55
K59	Other functional intestinal disorders	Digestive	284	9.71%	2.0	6.52
K92	Other diseases of digestive system	Digestive	149	5.09%	2.0	6.24
L03	Cellulitis	Skin	158	5.40%	2.0	7.01
F25	Schizoaffective disorders	Mental	229	7.83%	2.1	5.21
B96	Other bacterial agents as the cause of diseases classified to other chapters	Infectious	183	6.25%	2.2	6.64
E78	Disorders of lipoprotein metabolism and other lipidaemias	Endocrine	298	10.18%	2.4	6.17
E03	Other hypothyroidism	Endocrine	248	8.48%	2.4	5.96
F17	Mental and behavioural disorders due to use of tobacco	Mental	705	24.09%	3.0	5.08
J44	Other chronic obstructive pulmonary disease	Respiratory	148	5.06%	3.3	6.65
J18	Pneumonia, organism unspecified	Respiratory	246	8.41%	3.3	6.72
E66	Obesity	Endocrine	199	6.80%	3.8	6.01
E87	Other disorders of fluid, electrolyte and acid-base balance	Endocrine	205	7.01%	3.9	5.80
E86	Volume depletion	Endocrine	164	5.60%	3.9	6.98
A09	Diarrhoea and gastroenteritis of presumed infectious origin	Infectious	150	5.13%	4.0	5.93
N17	Acute renal failure	Genitourinary	228	7.79%	4.5	5.57
F20	Schizophrenia	Mental	219	7.48%	4.6	4.61

conditions after a patient was first diagnosed with schizophrenia.

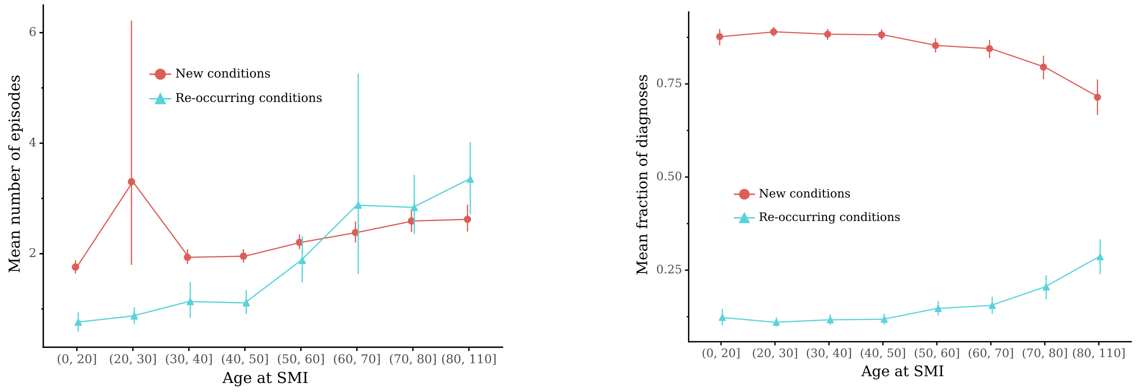

(a) (b)

Figure S5: (a) Mean frequency of a new condition and a re-occurring condition in post-SMI episodes. (b) Mean fractions of patients' diagnoses on new conditions and re-occurring conditions in post-SMI episodes, grouped by age at SIM diagnoses. Error bars show 95% CIs.

3 Post-SMI care patterns

In the main text, our results on multimorbidity progression reveal that a large number of new conditions occur in patients' post-SMI periods. However, these analyses have not taken the numbers of episodes on managing different conditions into account. Although the number of new conditions is large in post-SMI episodes, conditions that first appear in pre-SMI episodes, i.e., re-occurring conditions, can re-occur in post-SMI episodes multiple times. This raises a question is whether new or re-occurring conditions occur more frequently in post-SMI episodes. To explore this, we first measure the average frequencies of new and re-occurring conditions in each patient's post-SMI episodes by calculating the average edge weights from a patient node to her/his new and re-occurring condition nodes in the post-SMI network respectively. We find that a new condition appears on average in 2.38 ($SD = 27.26$) episodes in post-SMI periods, with a higher frequency than a re-occurring condition which appears in 1.37 ($SD = 6.97$) episodes on average.

By stratifying patients by the age at the first SMI diagnosis, we further find that re-occurring conditions appear in relatively more frequently in older individuals (Figure S5a). Moreover, by defining care focus as the fraction of diagnoses on new or re-occurring conditions in post-SMI episodes, we find that post-SMI care is dominated by managing new conditions which on average account for 87% of diagnoses (95% CI: 86.3%-87.7%) in a patient's post-SMI episodes. The high fraction of diagnoses on new conditions is widely observed across different age groups (Figure S5b). Moreover, the fraction of diagnoses on re-occurring conditions is increasing over SMI-diagnosis age, indicating that older individuals have higher intensity than younger individuals in managing re-occurring conditions in post-SMI care.

By stratifying patients by the SMI-diagnosis age in Figure S6, we further find that different age groups suffer from different new and re-occurring conditions in both pre- and post-SMI periods. For example, new mental health conditions, e.g., mental disorders due to use of tobacco ("F17"), are more prevalent in groups who were diagnosed with SMI at a younger age, while new physical health conditions such as hypertension ("I10") are more prevalent in groups with an older SMI-diagnosis age. Similar results are observed in re-occurring conditions in post-SMI periods, except that depressive episode ("F32") is more prevalent in re-occurring conditions than

in new conditions.

4 Network structures

Figure S7 shows correlations between disease node degree k and average degree of neighbors $\langle k_{nn} \rangle$ in the bipartite networks and those in their random counterparts. Figure S8 shows correlations between disease node degree k and the average number of second-order neighbors $\langle |N(N(k))| \rangle$ in the pre-SMI network and in its random counterparts.

To facilitate the analysis of relational structures among a particular set of nodes, bipartite networks can be compressed by one-mode projection, i.e., projecting a bipartite network to a one-mode network in which all nodes denote the same type of objects [1, 2]. For example, the original patient-diagnosis network can be projected to a disease co-occurrence network in which each node denotes a disease and two diseases are linked if they co-occur in the same patient. Then, $N(N(v))$ in the original bipartite network is direct neighbors of a disease node v in the projected network, i.e., the co-occurrence diseases of v. Likewise, we can project the bipartite network to a peer-to-peer network in which each node denotes a patient, and two patients are connected if they have the same conditions, leading to a one-mode network for patients. After this one-mode projection, we then measure the modularity by an attribute in the projected one-mode network to examine whether nodes with similar attributes tend to connect to one another [3, 4]. Table S6 shows the values of modularity by attribute observed in one-mode projected networks of patient nodes, where z-scores and p-values are computed based on 1,000 random networks. We find that patients with the same gender and ethnicity tend to connect to the same diseases, significant at $P < 0.001$ compared to random networks.

Table S6: Modularity by attribute observed in projected one-mode networks of patient nodes, as well their mean values and standard deviations (SD) in random networks. P values of observed results are assessed based on z-scores under a two-tailed hypothesis.

Network	Attribute	Observed	Mean	SD	z	P
Pre-SMI	Gender	2.61×10^{-2}	-5.07×10^{-4}	3.51×10^{-4}	75.87	<0.001
Post-SMI	Gender	1.58×10^{-2}	-4.69×10^{-4}	1.74×10^{-4}	93.31	<0.001
Pre-SMI	Ethnicity	2.27×10^{-2}	-6.25×10^{-4}	2.96×10^{-4}	78.76	<0.001
Post-SMI	Ethnicity	4.98×10^{-3}	-9.21×10^{-4}	1.54×10^{-4}	38.29	<0.001

References

[1] T. Zhou, J. Ren, M. Medo, Y.-C. Zhang, Bipartite network projection and personal recommendation, Physical review E 76 (4) (2007) 046115.

[2] M. Latapy, C. Magnien, N. Del Vecchio, Basic notions for the analysis of large two-mode networks, Social networks 30 (1) (2008) 31–48.

[3] M. E. Newman, Mixing patterns in networks, Physical review E 67 (2) (2003) 026126.

[4] M. E. Newman, Modularity and community structure in networks, Proceedings of the national academy of sciences 103 (23) (2006) 8577–8582.

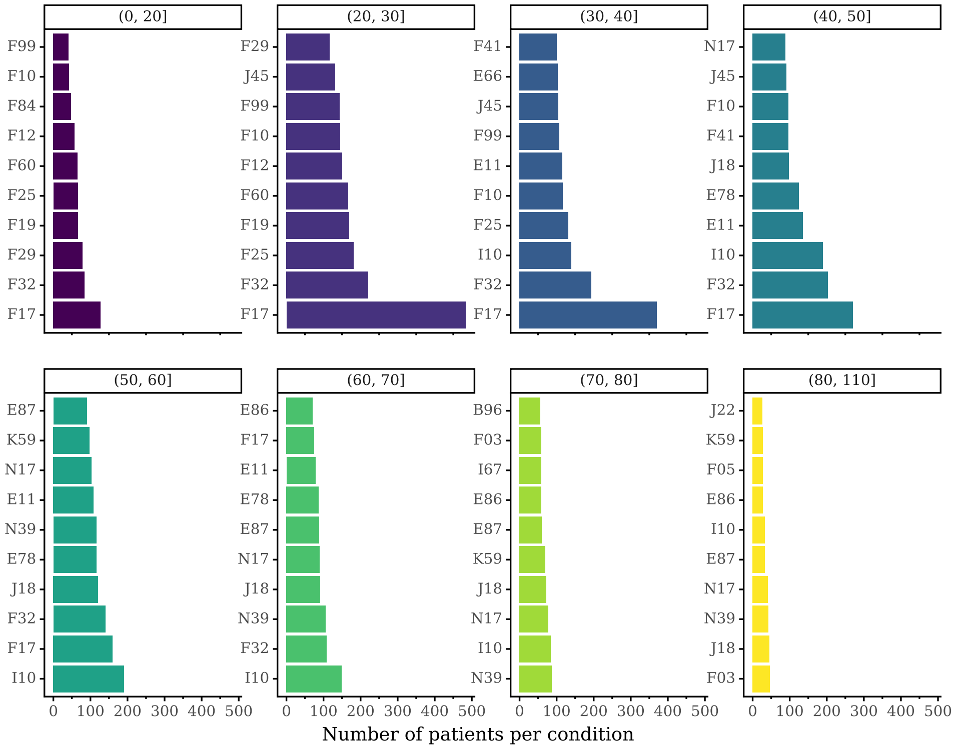

(a)

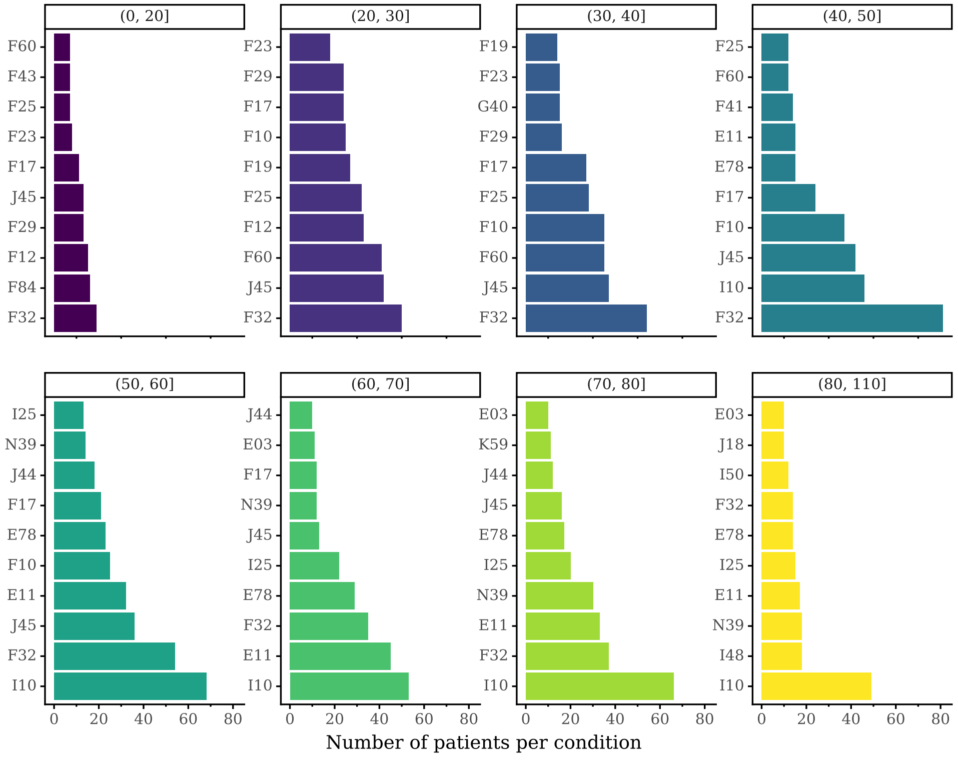

(b)

Figure S6: The top 10 prevalent diseases per SMI-diagnosis age group. (a) New conditions in post-SMI periods. (b) Re-occurring conditions in post-SMI periods.

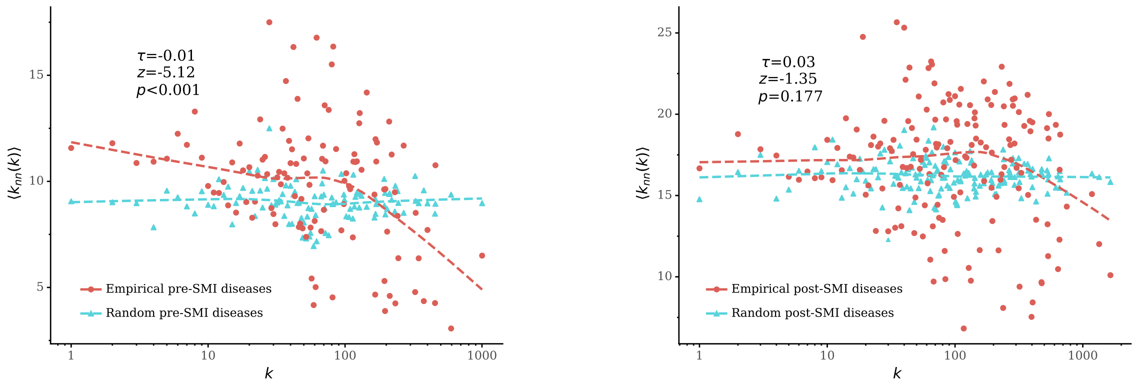

(a) (b)

Figure S7: Correlations between node degree k and average degree of neighbors $\langle k_{nn} \rangle$ in the bipartite networks and in their random counterparts. Dotted lines show the LOESS (Locally Estimated Scatterplot Smoothing) curves. The annotation marks the Kendall's τ coefficient between k and $\langle k_{nn} \rangle$ in the real-world networks, as well as its z-score and p-value compared to those measured in 1,000 random networks.

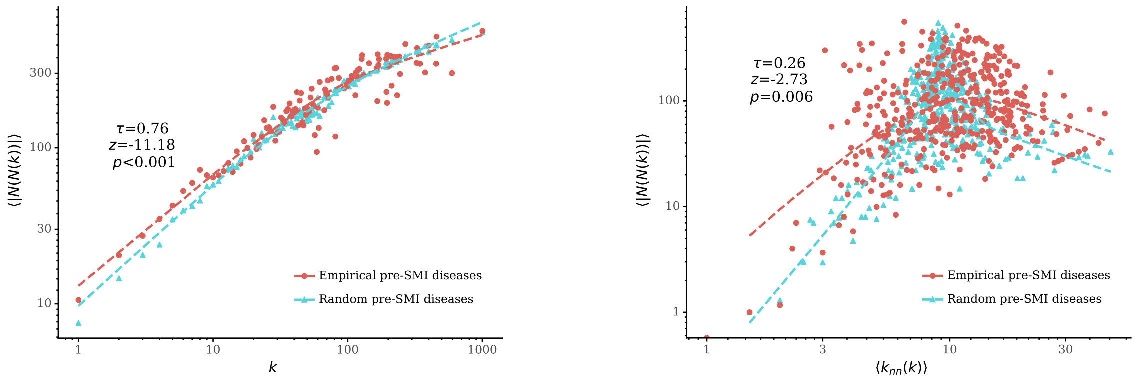

(a) (b)

Figure S8: (a) Correlations between node degree k and the average number of second-order neighbors $\langle |N(N(k))| \rangle$ in the pre-SMI network and in its random counterparts. (b) Correlations between the average degree of neighbors $\langle k_{nn}(k) \rangle$ and average number of second-order neighbors $\langle |N(N(k))| \rangle$ in the pre-SMI network. Dotted lines show LOESS curves. The annotation shows the Kendall's τ coefficient between two variables in the real-world networks, as well as its z-score and p-value compared to those in 1,000 random networks.
